# VIEW: An Assessment of Problem-Solving Style - 20 Years of Progress

**DOI:** 10.1177/00332941241249954

**Published:** 2024-04-26

**Authors:** Scott G. Isaksen, Christian Hoßbach

**Affiliations:** Department of Leadership and Organizational Behavior, 6281 BI-Norwegian Business School, Orchard Park, NY, USA; Department of Human Resources Management and Business Governance 197664 , Martin-Luther-Universitat Halle-Wittenberg, Halle, Germany

**Keywords:** Creativity, cognitive style, preferences, problem-solving style, individual differences

## Abstract

While the ability to solve complex problems creatively is among the most important skills for contemporary jobs, understanding individual differences how people prefer to engage in individual or collaborative problem-solving becomes increasingly important. VIEW an assessment of problem-solving style has been specifically designed to measure these preferences at the intersection of creativity and problem-solving. This article summarizes the progress that has been made in the past twenty years of research since the instrument was launched. The available evidence shows that the instrument meets contemporary standards of reliability and validity justifying its application in research and practice. Looking ahead, we conclude with promising areas for further developing the assessment and future research on problem-solving styles that addresses emerging phenomena such as collaborating across hybrid work environments or using artificial intelligence tools.

## Introduction

Problem-solving is essential to our everyday lives (Kirton, 2003) and solving complex problems creatively has become one of the most relevant capabilities to thrive and be successful in contemporary workplaces (Puccio, 2017). Thus, understanding individual differences how people engage in problem solving also becomes crucial information for organizations to enable people to develop their skill sets, improve collaboration with others, or drive change. There are many conceptions and measures of cognitive and problem-solving style (e.g., Armstrong et al., 2011; Kozhevnikov et al., 2014), as well as those that link to creativity (e.g., Kirton, 2003; Martinsen et al., 2020). Yet, most of these existing measures of style are uni-dimensional (Cools et al., 2014), making it difficult to capture the bigger picture of relevant style dimensions that are applicable for a phenomenon of interest in a particular context.

VIEW: An assessment of problem-solving style (VIEW) was introduced by Selby, Treffinger and Isaksen in 2004. They defined problem-solving styles as consistent individual differences in the ways people prefer to plan and carry out generating and focusing activities, in order to gain clarity, produce ideas, and prepare for action (Selby, 2004, p. 222). Since 2004, 26 books, manuals, and monographs; 83 published articles and chapters; 32 dissertations and theses; and other products have been published (Isaksen, et al., 2021a). The authors of VIEW also produced a series of technical resources that are publicly available to document the development, conceptual foundations, applications, and evidence regarding the assessment (e.g., Selby et al., 2021a, b, c). A variety of resources have been developed to qualify those who wish to use the assessment, as well as for applying the assessment.

The purpose of this article is to summarize the 20 years of research and development that has been accomplished, provide updated psychometric characteristics, and outline future research and applied issues for VIEW.

## Conceptual and Theoretical Foundations

The fundamental foundations of VIEW, detailed in Selby et al., (2021b), stem from two large domains of research and practice - namely, personality psychology of individual differences and creativity and creative problem solving. Within this intersection, the concept of problem-solving style builds on a unique integration of three main constructs related to individual differences that are relevant for engaging in a creative kind of problem-solving: learning style, cognitive style, and psychological type (Selby, 2004).

VIEW includes 34 items that assess three dimensions of problem-solving style. The Orientation to Change (OC) dimension includes three subscales or elements. These are defined in Table 1.

**Table 1.** The Dimensions of VIEW: An Assessment of Problem-Solving Style.

Orientation to Change - (18 items, 7-point response scale, scores range from 18 to 126. Each sub-scale contains 5 items with a range of 5 to 35.) Higher scores indicate a Developer preference.	
Explorers prefer to: Maintain energy by working on a wide array of tasks and envisioning the big picture; Welcome the freedom to create and follow one’s own rules and guidelines; and See deadlines as fluid and flexible.	Developers tend to: Maintain energy through persistence in working on a task and from the details of follow-through and implementation; Welcome rules and guidelines for how to complete a task. Seek, accept, and meet given deadlines.
*Sample Item:* When I am solving problems, I am a person who prefers making my own path.	*Sample Item:* When I am solving problems, I am a person who prefers following a familiar routine.
*Preference for Novelty* - Emphasize fundamentally new or original options; focusing on uniqueness	*Preference for Novelty* - Emphasize improvement of existing options; focusing on usefulness
*Structure and Authority* - Prefers autonomy and defining own approach, loose and permeable boundaries - assumes approval	*Structure and Authority* - Prefers detailed structure and direction or guidance, clear and defined boundaries - seeks approval
*Search Strategy* - Engages in broad search to explore without limits, open to a wide variety of resources - prefers breadth	*Search Strategy* - Engages in a focused search within limits, seeks targeted and relevant resources - prefers depth
Manner of Processing - (8 items, scores range from 8 to 56.) Higher scores indicate an Internal preference.	
Externals prefer to: Draw energy from interaction with other people; Share their thinking early, seeking input from others to refine and strengthen their thoughts before reaching closure; and may press to move quickly from ideas to action.	Internals prefer to: Draw energy from quiet reflection; Look to their own inner thoughts, considering ideas themselves before they are ready to share them with others; and prefers action that follows careful study.
*Sample Item:* When I am solving problems, I am a person who prefers thinking aloud ideas.	*Sample Item:* When I am solving problems, I am a person who prefers thinking quietly about ideas.
Ways of Deciding - (8 items, scores range from 8 to 56.) Higher scores indicate a task preference.	
Person-oriented deciders prefer to: Consider first the effect or impact of choices and decisions on people’s feelings; the need for harmony and positive interpersonal relationships.	Task-oriented individuals prefer to: Seek choices and decisions that are logical, sensible, and that can be justified objectively; Give the greatest weight to results and outcomes when making decisions.
*Sample Item:* When I am solving problems, I am a person who prefers sympathetic and caring decisions.	*Sample Item:* When I am solving problems, I am a person who prefers fair and just decisions.

VIEW has shown its ability to identify preferences for learning and applying various aspects of creative problem solving (CPS). Some problem-solving style assessments base their measurement on a specific model of CPS (e.g., Basadur et al., 2014; Puccio & Grivas, 2009). VIEW is agnostic to any specific model of CPS as this would ‘muddle’ style with process which is something to be avoided (e.g., Kirton, 2003). Rather, the styles identified within VIEW have shown relationships to reported preferences for learning and using specific tools, guidelines, stages, and components of CPS by using independent measures. Other studies included within this article highlight relationships to other aspects of creativity. There is ample empirical support for the conceptual foundations for VIEW providing evidence for its validity that will be detailed below.

## Descriptive Statistics

Since the initial launch of the VIEW assessment, a database has been maintained on those who completed the measure and agreed to have their results included. The source of this data is derived by those who are qualified to use the assessment, so this is not a randomized sample, but rather a collection of numerous samples of convenience. All data has been collected in conformance with the APA’s ethical policies, as well as those of the Data Protection regulations.

The current master database for VIEW used for these analyses includes 64,880 subjects. Based on 62,227 subjects who provided age data, the mean age was 33.3 (SD = 14.8; range, 10–94). The database includes 29,856 male respondents (46.5%), 34,309 female respondents (55%), and 720 respondents (1.1%) who declined to state their gender. Table 2 summarizes several important descriptive statistics for each of VIEW’s three dimensions: Orientation to Change (OC), Manner of Processing (MP), and Ways of Deciding (WD), and the three subscales of OC, Novelty (NV), Structure and Authority (SA), and Search Strategy (SS).

**Table 2.** Descriptive Statistics for VIEW.

Statistic	Orientation to Change (OC)				Manner of Processing	Ways of Deciding
	Total OC	NV^a^	SA^b^	SS^c^		
Mean	74.88	19.33	20.29	21.87	29.55	35.21
Standard Deviation	15.7	5.58	5.4	5.5	9.15	8.3
Median	75	19	20	22	29	35
Mode	72	20	20	23	32	32
Minimum	18	5	5	5	8	8
Maximum	126	35	35	35	56	56
Skewness	−0.205	−0.006	−0.026	−0.367	0.232	−0.14
Kurtosis	0.094	−0.335	−0.221	−0.114	−0.217	−0.172
Alpha	0.862	0.872	0.871	0.872	0.856	0.856
Standard Error of the Mean	5.83	1.99	1.94	1.97	3.47	3.15

Note. *N* = 64,880.

^a^Preference for Novelty.

^b^Structure and Authority.

^c^Search Strategy.

### Intercorrelations Among VIEW’s Dimensions, Age, and Gender

Table 3 presents the intercorrelations among VIEW’s three dimensions, and the correlations of both age and gender with each of the three VIEW dimensions. The intercorrelations among VIEW’s three dimensions are of very low magnitude. As expected, all three of the subscales correlate strongly and significantly with the overall OC dimension.

**Table 3.** Correlations Among VIEW Dimensions.

Variable	1	2	3	4	5	6	7	8
1. Orientation to Change	1.00	0.12	0.11	0.83	0.80	0.74	−0.09	0.12
2. Manner of Processing		1.00	0.13	0.18	0.05	0.04	−0.05	0.02
3. Ways of Deciding			1.00	0.03	0.00	0.25	0.09	−0.27
4. Preference for Novelty				1.00	0.52	0.45	−0.09	0.10
5. Structure and Authority					1.00	0.41	−0.19	0.18
6. Search Strategy						1.00	0.10	−0.02
7. Age							1.00	−0.21
8. Gender								1.00

Note. *N* = 64,880. All Correlations are significant at the *p* < .01 level, except between 3 and 5.

The correlations of VIEW’s dimensions with age or gender are also negligible. Although these correlations are statistically significant (probably by virtue of the large sample size), note that the magnitude of the relationship is very weak (accounting for a very small amount of the variance). The relationship between gender and the Ways of Deciding dimension is somewhat stronger, but still accounts for only 10% of the variance; it suggests a slight tendency for female subjects to have a Person-oriented preference and for male subjects to have a Task-oriented preference. This result is similar to findings for other similar inventories in its direction, as well as in its modest magnitude (e.g., Myers et al., 1998).

### VIEW Translations

We have been actively engaged in research and development to make VIEW available in languages other than English. We follow the translation procedures outlined by the American Psychological Association (2003), and Geisinger (2003). The translation process starts with creating a forward translation from English to its target language. The next step is to conduct a back-translation from this initial translation into English and make adjustments to ensure linguistic relativity. Then a research edition is prepared and applied to allow for analysis of item performance, reliability, and factor structure. Modifications are made to ensure close fit to the original measure. In most cases, additional research is conducted to examine if the new translation performs similarly to the English version.

On-line editions of VIEW are currently available in Dutch, Chinese, Korean, French, German, Japanese, Spanish, and Norwegian. Table 4 summarizes the current data regarding the internal consistency of the completed translations. The table includes results for the three main dimensions of VIEW along with the three subscales or elements contained within the OC dimension. Additional research evidence supporting the Dutch edition has also been completed (Isaksen et al., 2010). Similar research was conducted in order to create the German translation and were reported in Hoßbach and Lange (2015), and for the Chinese translation in Chiu (2008).

**Table 4.** Cronbach’s Alpha Values for VIEW Translations.

Translation	*N*	Orientation to Change (OC)				Manner of Processing	Ways of Deciding
		Total OC	NV^a^	SA^b^	SS^c^		
Chinese	1164	.82	.81	.81	.81	.81	.81
Dutch	2641	.87	.87	.86	.87	.86	.86
French	459	.80	.80	.51	.66	.80	.72
German	382	.87	.87	.86	.86	.86	.86
Japanese	369	.84	.84	.83	.84	.83	.83
Korean	658	.87	.87	.87	.87	.87	.86
Norwegian	683	.87	.87	.87	.86	.87	.86
Spanish	456	.85	.84	.84	.85	.85	.85

^a^Preference for Novelty.

^b^Structure and Authority.

^c^Search Strategy.

As with any translation effort, we continue to learn more about cultural differences and language nuances that will guide future improvements and development. For example, as we have developed the three sub-scales of the Orientation to Change dimension, there appear to be challenging nuances particularly on the Structure and Authority element. Our future efforts will focus on increasing sample sizes, gathering additional reliability and validity data, and gaining a better understanding of statistical differences. We will also continue to investigate the feasibility of translations into other languages.

## Reliability

The data from our developmental studies indicated that VIEW meets the customary expectations regarding reliability to support use in research and training contexts, in relation to both *stability* and *internal consistency.* Additional detail regarding both stability and internal consistency results throughout VIEW’s development is available in Selby et al., (2021a) and Isaksen et al. (2021b).

### Stability

Isaksen et al. (2021b) reported the results of four stability studies. The first reported test-retest results over a one-month interval, was carried out with 48 middle school students and nine adults. The correlations were .90, .60, and .65 for the OC, WD, and MP dimensions respectively. In another study of stability involving 23 adults over a one-month period, the correlations were .85, .80, and .77 respectively. Nineteen subjects completed VIEW again after two months. The two-month stability correlations were .93 for the OC dimension, .93 for MP, and .84 for the WD dimension.

An additional test-retest study was carried out with 49 undergraduate students in a teacher education program in an urban setting in the Northeastern United States. The two-month test-retest correlations were: .83 for OC, .84 for MP, and .75 for WD. We have also gathered data for a 12-month, test-retest reliability study. For an adult sample (*N* = 52), the 12-month test-retest reliability coefficients were: Orientation to Change, *r* = .74; Manner of Processing, *r* = .83, and Ways of Deciding, *r* = .81. These data provide support for the claim that scores on the VIEW instrument are stable, and that the instrument meets or exceeds the customary standards and expectations for reliability over time.

### Internal Consistency

We examined the internal consistency of VIEW’s three dimensions using Cronbach’s coefficient Alpha. The coefficient Alpha results for the current master database (*N* = 64,880) were .86 for OC, .86 for MP, and .86 for WD. The coefficient Alpha for the three sub-scales of OC were .87 for Novelty, .87 for Structure and Authority, and .87 for Search Strategy. These results exceed the generally accepted criterion that internal consistency should be > .70, and therefore support our claim for the internal consistency of VIEW.

## Validity

Demonstrating validity, is an on-going process, not an “event” that can be established definitively in a single study or a specific set of results. Therefore, validation of VIEW, like any other instrument, requires an ongoing program of research by the developers and the active contributions of many other researchers over a period of years; it is also influenced by the goals and actions of those who use the instrument and its results in various contexts. Our validation efforts, including both quantitative and qualitative procedures, have been ongoing and continuously expanding over the two-decades-long history of VIEW’s availability. Below is a discussion of those efforts across five interrelated sources of evidence comprising a contemporary understanding of validity (AERA, APA, & NCME, 2014).

### Evidence Based on Test Content

This first source of evidence relates to the relationship of the VIEW items to its theoretical and conceptual rationale. Selby et al. (2021a & b) provided a detailed description of the conceptual and theoretical foundations of the VIEW assessment, as well as the many stages of item development and testing. At each stage, statistical item analysis was performed resulting in the rewording or removal of items that did not perform well.

Studies have provided evidence supporting VIEW test content. Houtz (2002) studied the relationship between VIEW dimensions and problem-solving strategies. Selby et al. (2003) examined construct validity of VIEW by examining MBTI type and personality characteristics. The linkages between VIEW and preferences for, and use of, specific creative problem-solving methods and tools has also been studied (Isaksen & Geuens, 2007; Schoonover & Treffinger, 2003). The profile of relationships within all these studies was consistent with VIEW theory and content.

Treffinger et al. (2008) reported on more than five decades of research and development on making the creative problem-solving process and tools accessible across a wide range of ages and contexts. They concluded that recent evidence indicated that when individuals, in both school and corporate settings, understand their own style of problem solving, they learn and apply process tools more effectively, and when teams appreciate the styles of their individual members, their problem-solving efforts are enhanced. They proposed that evidence supports the conclusion that individual style differences provide an important key to understanding the interaction of person, process, product, and press when managing change.

### Evidence Based on Response Processes

This source of evidence relates to the ‘fit’ between the construct and the detailed nature of the performance or responses provided by those who complete the assessment. There are many conceptions and measures of cognitive and problem-solving style (e.g., Kozhevnikov et al., 2014), as well as those that link to creativity (e.g., Martinsen et al., 2020). Kogan (1973; 2017) and Messick (1984; 1996) made clear distinctions between styles as propensities and abilities or capabilities. Abilities tend to be value directional (more is better) whereas styles tend to be value differentiated (all styles have value - but may have more in certain circumstances). They also pointed out that some styles may be intimately intertwined with ability. For example, field dependence versus independence has been considered a cognitive style, but it shows a very clear relationships with a variety of level-oriented capabilities (e.g., Giancola et al., 2022; Páramo & Tinajero, 1990). Other styles may be more clearly aimed at preference.

Kirton (1978; 2003) sharpened the level-style distinction by positing that there should be no relationship of his measure of style (KAI) and level of creativity. This led to a shift from asking, “How creative are you?” to the challenging question, “How are you creative?” Our efforts moved beyond looking at *level* of creativity to considering *style* of creativity (varied ways of expressing and applying creativity). The evidence supporting Kirton’s assertion has been equivocal (e.g., Isaksen & Puccio, 1988; Kaufmann, 2004). Kirton’s measure presents adaptors and innovators as a value-neutral continuum, yet research has demonstrated that individuals place implicit value on the innovator style of creativity (Puccio & Chimento, 2001; Ramos & Puccio, 2014).

From the origins of VIEW’s development, the primary goal was to shift the focus of attention from responses that emphasized level of creativity to response processes that illuminate the person’s style of creating, solving complex problems, and managing change. The development of VIEW emphasized the importance of maintaining a clear distinction between level and style in response processes, increasing our understanding and appreciation of diverse styles of creativity and their contributions to understanding, defining, assessing, and nurturing creativity, and improving creative abilities (Isaksen, 2004).

The evidence, thus far, shows that VIEW assesses an individual’s style preference, and not level of creative capability or intelligence. Houtz and Selby (2009) examined the relationship between VIEW and two measures of creative productivity and found no significant relationships. Woodel-Johnson et al. (2012) found no relationship between VIEW and verbal and figural forms of the Torrance Test of Creative Thinking. Isaksen et al. (2016) found no significant relationships between the reasoning/intelligence scale of the Cattell 16PF and VIEW. Finally, Robertson (2017) examined the effects of problem-solving style on cognitive load of participants while engaged in a writing task. The judges who rated the creativity of the writing task showed no relationship to the VIEW dimensions.

Evidence based on response processes also involves respondents’ perceptions and self-awareness of style. In two early stages of the development process of the VIEW instrument, we gathered qualitative data regarding subjects’ responses to the VIEW inventory and their perceptions of the accuracy and clarity of their results. In studies reported by Isaksen et al. (2021b), participants reported a high degree of agreement that their overall scores on VIEW agreed with their own personal assessment. Selby (2004) and Isaksen and Kaufmann (2013) conducted similar studies and reported a high level of agreement between actual VIEW scores and personal estimates.

Isaksen et al. (2016) investigated response styles and acquiescence on a personality inventory among participants in their study, as they completed both VIEW and the 16PF (Cattell et al. 1970). The 16PF includes three response style indicators. The Acquiescence scale of the 16PF measures the tendency to answer “true” to an item regardless of its content. The results for this sample indicated that the VIEW respondents did not respond randomly or indecisively. The Impression Management scale is essentially a social-desirability scale with high scores indicating socially desirable responses and low scores reflecting a willingness to admit to undesirable characteristics. The resulted indicated that subjects completing VIEW did not respond in a socially desirable fashion. The Infrequency scale is designed to indicate if a respondent answers a relatively large number of responses in a way that is different from most people. Results indicated that those completing VIEW did not indicate a relatively uncertain response orientation.

### Evidence Based on Internal Structure

We have gathered evidence supporting the claim that the internal structure of VIEW is consistent with the three dimensions it purports to represent. In three separate stages of VIEW’s development, we conducted exploratory factor analyses to evaluate the extent to which our three hypothesized factors would be supported by the evidence. Table 5 presents the summary results of the factor analysis for the current master data set using principal components analysis.

**Table 5.** Factor Analysis of VIEW Items.

VIEW Item	Component		
	1	2	3
OC-1	.641		
OC-2	.383		
OC-3	.467	.312	
OC-4	.553		
OC-5	.522		
OC-6	.639		
OC-7	.590		
OC-8	.543		
OC-9	.624		
OC-10	.722		
OC-11	.629		
OC-12	.733		
OC-13	.435		
OC-14	.529		
OC-15	.515	.324	
OC-16	.638		
OC-17	<.30		
OC-18	.369		
MP-1			.748
MP-2			.710
MP-3			.740
MP-4			.711
MP-5			.560
MP-6			.707
MP-7			.720
MP-8			.677
WD-1		.675	
WD-2		.564	
WD-3		.568	
WD-4		.700	
WD-5		.649	
WD-6		.760	
WD-7		.645	
WD-8		.739	

Note. *N* = 64,880. Extraction Method: Principal Component Analysis with Varimax rotation and Kaiser Normalization (converged in 5 iterations). Factor loadings > .30 displayed.

**Table 6.** Factor Analysis of the OC Dimension.

VIEW Item	Component		
	1	2	3
OC-1	.520	.365	
OC-10	.588	.508	
OC-17		.327	
OC-2 (SS)			.658
OC-3 (SS)			.717
OC-13 (SS)			.650
OC-14 (SS)			.528
OC-15 (SS)			.655
OC-4 (NV)		.735	
OC-6 (NV)		.658	
OC-9 (NV)		.726	
OC-11 (NV)	.339	.516	
OC-12 (NV)	.465	.617	
OC-5 (SA)	.669		
OC-7 (SA)	.616		
OC-8 (SA)	.622		
OC-16 (SA)	.671	.316	
OC-18 (SA)	.468		.380

Note. *N* = 64,880. Extraction Method: Principal Component Analysis with Varimax rotation and Kaiser Normalization (converged in 5 iterations). Factor loadings > .30 displayed.

The Scree plot illustrated Eigen values for three main factors ranging from 3.7 to 6. Yet, three other factors resulted in values over one. Selby (2013) investigated the extent to which, as subjects’ overall preference for either the Explorer or Developer style became more well defined, their scores on each of the three OC elements (Novelty, Structure and Authority, and Search Strategy) would tend to move to either end of the OC dimension while those with moderate OC preferences might score on the other side of the mean. Thus, those with a moderate OC preference for Developer might prefer an Explorer’s approach to one of the three elements. The researcher tested this using data from 867 respondents. The results confirmed expected patterns.

Further investigation of the OC dimension, containing 18 items, led to the development of three subscales or elements. The results are displayed in Table 6. Each of these sub-scales focused on a singular sub-factor. One for individuals’ preference for Novelty. One for preferences surrounding Structure and Authority. The third for Search Strategy. Those five items loading most heavily on each of these sub-factors were selected for inclusion into each subscale.

Two previous studies examined the confirmatory factor analysis (CFA) of VIEW on earlier versions of the database. Proestler and Vazquez (2011) reported a good fit to the model (*GFI* = .984, *CFI* = .984, *RMSEA* = .054, *N* = 25,000). Isaksen and Aerts (2011) found an adequate fit to the measurement model (*GFI* = .86; *AGFI* = .85, *NFI* = .82, *RMSEA* = .06). CFA results on the current database provide adequate support for the three main dimensions (*TLI* = .791; *CFI* = .816; and *RMSEA* = .061, *N* = 19,065), but better fit for including the three sub-scales of Orientation to Change (*TLI* = .863; *CFI* = .883; and *RMSEA* = .05) within the measurement model.

### Evidence Based on Relationships With Other Variables

We have studied correlations between scores on the VIEW instrument and several other measures that represent the theories and models that influenced us in designing and developing our instrument representing current evidence of concurrent validity.

In line with VIEW’s conceptual foundations, we found relationships of VIEW’s dimensions and other measures of style. Isaksen et al. (2021b) reported three studies conducted on the relationship between VIEW and the Dunn and Dunn learning style model (Dunn et al., 1993). Two additional published studies (e.g., Delcourt, 2013; Woodel-Johnson, 2010) provided profiles of learning style that are consistent with the VIEW model. Isaksen et al. (2021b) reported on two studies examining the relationship between Kirton’s measure of cognitive style (KAI, Kirton, 2003) and VIEW. Isaksen and Kaufmann (2013) reported an additional study of the relationships between the KAI and VIEW and found significant relationships that were consistent with the model. Isaksen et al. (2021b) reported results of the relationship between VIEW and the MBTI as a measure of psychological type (Myers et al., 1998). Again, relationships were in the expected directions.

Studies have been conducted to examine the personality underpinnings of VIEW styles. Houtz et al. (2010) examined the relationship between VIEW problem-solving styles and multicultural personality dispositions using the Multicultural Personality Questionnaire (Van der Zee & Van Oudenhoven, 2000). Landers et al. (2012) studied the relationships between VIEW and a 40-item self-checklist of personal characteristics. Isaksen et al. (2016) conducted a study to examine the deeper personality foundations of VIEW using the Cattel 16 Personality Factor Questionnaire (Cattell et al., 1970). All three studies provided personality profiles that were consistent with the VIEW dimensions. However, it is important to note the magnitude of these relationships does not suggest a redundant overlap of style and personality as critiqued by von Wittich and Antonakis (2011), but rather moderate patterns of relationships that are in line with VIEW’s conceptual foundations.

The relationships between VIEW and a variety of other constructs have been studied over the past 20 years. Houtz et al. (2007) studied the relationship between motivational attribution patterns of success and failure in problem solving. Johnson et al. (2014) studied career orientations and relationships with VIEW. Neyen et al. (2017) studied how VIEW related to parenting styles. Maghan (2017) examined the relationships between VIEW and coping styles. Gashi (2020) studied the relationships between VIEW styles and conflict management modes. The results of these studies show patterns of relationships that are in accordance with VIEW’s conceptual and theoretical foundations.

### Evidence Based on Consequences and Uses

Evidence of the validity of VIEW also comes from documentation of the instrument’s effective application across a variety of goals, purposes, and situational contexts. VIEW has been shown to be a powerful and valuable tool, for adolescents and adults, in many different organizations, and for a variety of purposes. This section presents an overview of the successful applications and impacts of VIEW across ages, places, and settings.

Early applications of VIEW were aimed at learning and instruction. Treffinger and Schoonover (2003) reported on an application of VIEW in an educational setting involving curriculum development for problem-solving based learning. They found that informing the group of curriculum developers about their style helped them remove bias in the exercises they were creating. Shaw et al. (2009) applied VIEW with preservice teachers and were able to link their styles to principles of learning, teaching, and problem solving. Other studies have also demonstrated the usefulness of VIEW in helping educators provide instructional differentiation (Delcourt et al., 2015; Southwell, 2015; Treffinger et al., 2013a).

Another practical application of VIEW has been aimed at improving teamwork and creative collaboration. McCoy and Houtz (2011) investigated VIEW style and creative productivity of college student teams and found appropriate differences style-related differences in the output of their projects.

Treffinger et al. (2013b) discussed the uses of problem-solving style and process tools to optimize leadership and team performance. They argued that when team members understand their problem-solving style along the three dimensions assessed by VIEW and the interaction of style with CPS components and stages, they will increase their effectiveness in meeting creatively the challenges posed by rapid change. Schroth et al. (2015) conducted an experiment that provided clear evidence to support the assertion made by Treffinger and colleagues. Further support was provided by Main et al. (2019), who conducted an experimental study comparing the performance of team members who received feedback on VIEW with a control group. They reported a significant difference in creative problem-solving performance for those teams receiving VIEW feedback.

Isaksen and Tidd (2006) devoted a chapter entitled *Teamwork for Transformation: Applying VIEW to help make teams productive* within the context of leadership for organizational transformation and growth. They integrated VIEW dimensions within the dynamic model for group development to illustrate how leaders can prepare groups and teams for change. Some studies have linked various aspects of leadership to VIEW (e.g., Delgado, 2019; Tamvakologos, 2018). Others have demonstrated the value of applying VIEW to help organizations prepare for change and transformation (e.g., Lofquist & Isaksen, 2019).

## Summary and Future Pathways

The first 20 years of VIEW have provided a rich foundation, and adequate support for the psychometric quality, reliability, and validity of the assessment. As with any measure, the research and development journey must continue to build and expand upon this foundation. VIEW is designed to assess problem-solving style at the individual level of analysis, but has relevance to teams, as well as organizations. We need to conduct further investigation into:

### Style Preference, Coping, and Actual Problem-Solving Behavior

VIEW is a self-report measure that assesses an individual’s preference for problem-solving behavior. Although we have gathered some evidence to indicate that these preferences differentiate some aspects of actual behavior, more work needs to be done.

Individuals who interact with others who hold very different problem-solving styles or must face tasks demands that differ from their preferences, generally face three options. The first, and least desirable, is to experience clash or personal tension as they struggle with conflict or even hostility. The second is to cope or chose to behave in a way that is not consistent with their individual preferences. The third is to find ways to collaborate with others who may have a better style-task fit.

Coping takes energy, can create stress and tension and if demanded in extreme amounts over long periods it can have negative effects on mental and physical health (Cooper & Quick, 2017). There are a variety of methods to mitigate the cost of coping, one of which is the learning and applying of CPS. Contemporary approaches to CPS include a broad and balanced range of diverging (generating) and converging (focusing) tools for which VIEW style preferences are already known. Having access to tools and techniques may reduce the costs of coping when individuals face challenges outside their comfort zone. This involves a certain level of metacognition (Isaksen, 2020). Further, when individuals engage in creative collaboration, social metacognition can help groups engage in planning their approach to problem solving (e.g., Isaksen, 2023). This can involve deciding which tools to use with whom, when and how. Further inquiry is needed to examine how different style preferences play a role in these processes.

Some styles may be better suited to flex their behavioral responses to challenges. For example, Bakken et al. (2023: p. 1) found that: “Our results indicate that flexible style preferences boost the effect of cognitive ability, while strong preferences for a single style may entrench even those with high cognitive abilities.” Future research should examine the effects of moderate style preferences on the ability to maintain a flexible posture when engaged in creative problem-solving behavior.

Finally, there are numerous other individual factors that should be examined when it comes to actual CPS behavior and the cognitive mechanisms that drive successful outcomes when addressing open-ended and ill-defined problems. For example, further work can be done to better understand the potential linkages between problem-solving style and cognitive fixation (e.g., Gauselmann et al., 2023; Wang et al., 2023), cognitive load (e.g., Chen et al., 2023; Paas et al., 2010), and cognitive biases (e.g. Acciarini et al., 2021; Tversky & Kahneman, 1974), among others.

### The Level-Style Distinction

The authors and qualified users of VIEW share a commitment to provide value-neutral style preference information to end users. The extent to which VIEW style dimensions are independent from various other creative level or capability measures needs further examination to determine how the level-style distinction applies (Isaksen, 2004; Kirton, 1978).

### Effects of Diversity

Hammerschmidt (1996) studied the effect of style diversity on team performance and found that various style combinations had significant effects on success rates. Further VIEW research should deal with examining effects of diverse problem-solving styles on a variety of creative and innovative processes and methods. What might be the optimum level of problem-solving style diversity within teams when facing real problems and challenges and under what circumstances?

### Interaction With Work Environment

Kozhevnikov et al. (2014) see cognitive styles as environmentally sensitive individual differences. Some exploratory research has already examined the relationships between VIEW and organizational work environment (e.g., Hoßbach, 2019; Isaksen & Aerts, 2011), yet further work should be done to improve our understanding of how different problem-solving styles differ in relation to the facets of the work environments that are supporting or inhibiting their preferred approach to engaging in creative problem-solving. This work would have important implications for leadership, as well as person-organization and person-environment fit (Kristof-Brown et al., 2023; van Vianen, 2018).

This kind of inquiry also has implications for recent technological developments such as the trend toward hybrid working (Alfes et al., 2023) and applications of artificial intelligence (Jia et al., 2024). How does individual style preferences influence the way in which people design their individual work environments by taking advantage of the increased autonomy about where and when they work? How can people be enabled to find out where, when, and how they can work most creatively to make informed decisions within the broad variety of possible choices they can make? How do style differences influence if and how people are willing to use artificial intelligence tools when they engage in creative problem-solving? How does this influence how they cope with different task demands such as focusing on the elements of a task that align more with people’s style preferences and outsource other elements to AI? Style preferences provide a promising lens for better understanding how these emerging opportunities might be managed effectively.

### Cross Cultural Comparisons

We assume that problem-solving style is a ubiquitous, cross-cultural construct. The translation process for VIEW has highlighted the importance of linguistic relativity - that speakers of different languages may think differently (e.g., Casasanto, 2016). Further research on VIEW should examine the extent to which the style dimensions are actually and behaviorally similar (or different) across cultures.

## Conclusion

The first 20 years of research and practice has shown that VIEW possesses adequate psychometric characteristics, as well as reliability and validity evidence. Further research is warranted in numerous directions outlined above. Further, the growing community or practitioners can be a productive source to identify additional issues and questions regarding the future applications of this assessment.

## Data Availability

Data sharing not applicable to this article as no datasets were generated or analyzed during the current study.
